# Posterior instrumentation combined with anterior debridement and reconstruction using allogenic strut bone for the treatment of children with multilevel lumbar spinal tuberculosis: minimum 5-year follow-up

**DOI:** 10.1186/s12891-022-06006-0

**Published:** 2022-12-02

**Authors:** Jingyu Wang, Xueying Zhang, Yi Zhang, Guohua Lv, Xiaobin Wang, Jing Li

**Affiliations:** 1 Department of Spine Surgery, Spinal Deformity Center, The Second Xiangya Hospital of Central South University, Changsha, 410011 Hunan China; 2 Department of Neurology, The First Affiliated Hospital of Xinxiang Medical University, Weihui, 453100 Henan China

**Keywords:** Allogenic strut bone, Children, Kyphosis, Lumbar spinal tuberculosis, Multilevel

## Abstract

**Objectives:**

To evaluate the clinical outcomes of one-stage posterior instrumentation combined with anterior debridement and reconstruction using allogenic strut bone for the surgical treatment of multilevel lumbar spinal tuberculosis in children younger than 10 years of age with at least 5 years of follow-up.

**Methods:**

A total of 16 children with multilevel lumbar spinal tuberculosis who underwent one-stage posterior instrumentation combined with anterior debridement and reconstruction using allogenic strut bone were enrolled from January 2003 to January 2017. Among them, 6 were females and 10 were males with an average age of 6.9 ± 2.2 years (range 3–10 years). Patients’ clinical outcomes, including C-reactive protein (CRP), erythrocyte sedimentation rate (ESR), kyphosis angle, and neurologic function, were assessed before and after surgery. *P* < 0.05 was considered statistically significant.

**Results:**

The average follow-up was 7.8 ± 2.4 years. CRP and ESR of all patients returned to the normal range within 1 year. Compared with preoperative neurological deficits, postoperative and final follow-up neurological deficits improved significantly by grades 0.9 and 1.6, respectively. No instrumentation failure occurred, and all patients achieved solid bone fusion. The preoperative kyphosis angle was 29.9 ± 8.1°, which decreased significantly to 5.9 ± 2.6° postoperatively. There was a mild loss (2.5°) and the kyphosis angle was 8.4 ± 2.9° at final follow-up, with an overall correction rate of 71.3%.

**Conclusion:**

One-stage posterior instrumentation combined with anterior debridement and reconstruction using allogenic strut bone is a safe and effective procedure for children with multilevel lumbar spinal tuberculosis. This approach facilitates the removal of lesions and decompression of the spinal cord and is effective in restoring spinal stability, correcting kyphosis, and preventing deterioration of the deformity.

## Introduction

Tuberculosis (TB) is an ancient global epidemic and remains one of the major causes of death worldwide. The latest global TB report shows that there are approximately 10 million new TB cases each year, with children accounting for 11% [1]. Spinal TB, a common form of extrapulmonary TB, accounts for about half of skeletal TB and has a high prevalence in the lumbar region [2]. *Mycobacterium tuberculosis* often invades the anterior spinal column, while the posterior structures are usually intact, thus predisposing the spine to kyphosis. Due to the abundant blood supply of the cartilage endplates and annulus fibrosus in children, *Mycobacterium tuberculosis* can spread easily between different segments through the vessels, resulting in erosion and destruction of multilevel vertebrae. Besides, prevertebral fascia and periosteum are loosely connected to the vertebrae, leading to extensive abscesses that diffuse between these potential spaces [3]. Compared with adults, special anatomical and physiological characteristics of children make the spine susceptible to more lesion-involved segments, which may result in cardiopulmonary dysfunction, serious spinal deformity, nerve dysfunction, or even paralysis [4, 5].

Standardized application of anti-TB drugs is the cornerstone for the treatment of TB. Although chemotherapy can control the disease in some patients with spinal TB, residual kyphosis cannot be corrected. Worse still, because the posterior spinal column of children still has growth potential, the deformity may progress even after the lesion has healed. Rajasekaran et al. [6] found that spinal deformities aggravated in 39% of children with cured spinal TB during the growth spurt, and about 3% had a kyphotic deformity greater than 60°. Therefore, surgery is important for correcting spinal deformity, preventing aggravation of kyphosis, and saving neurological function. Although various surgical procedures have been reported for the treatment of spinal TB in children, the optimal surgical approach remains controversial [7]. Additionally, there is no consensus on the choice of graft material for anterior column reconstruction, especially for children with long segment defects.

To our knowledge, only a few studies have reported the outcomes of one-stage posterior instrumentation combined with anterior debridement and reconstruction using allogenic strut bone for the surgical treatment of multilevel lumbar spinal TB in children younger than 10 years of age. The present study sought to evaluate the clinical efficacy of this strategy with at least 5 years of follow-up.

## Methods

### Patient population

From January 2003 to January 2017, patients younger than 10 years old, who underwent surgical treatment for lumbar spinal TB at the Second Xiangya Hospital of Central South University were initially reviewed retrospectively. The indications for surgery were as follows: (1) extensive paravertebral abscesses; (2) large sequestrum and cavity; (3) spinal instability, pathological dislocation, and kyphosis deformity due to severe bone destruction; (4) neurological deficits; and (5) the above conditions were ineffective with conservative treatment, and even deteriorate.

The inclusion criteria were: (1) active lumbar TB confirmed by clinical manifestations, laboratory tests, and pathological examination; (2) at least two continuous motion segments involved; (3) single-stage posterior instrumentation and anterior debridement and reconstruction using allogenic strut bone; (4) complete medical records and imaging data; (5) follow-up for at least 5 years. Exclusion criteria include (1) active pulmonary TB; (2) posterior or anterior-only approach.

**Fig. 1:**
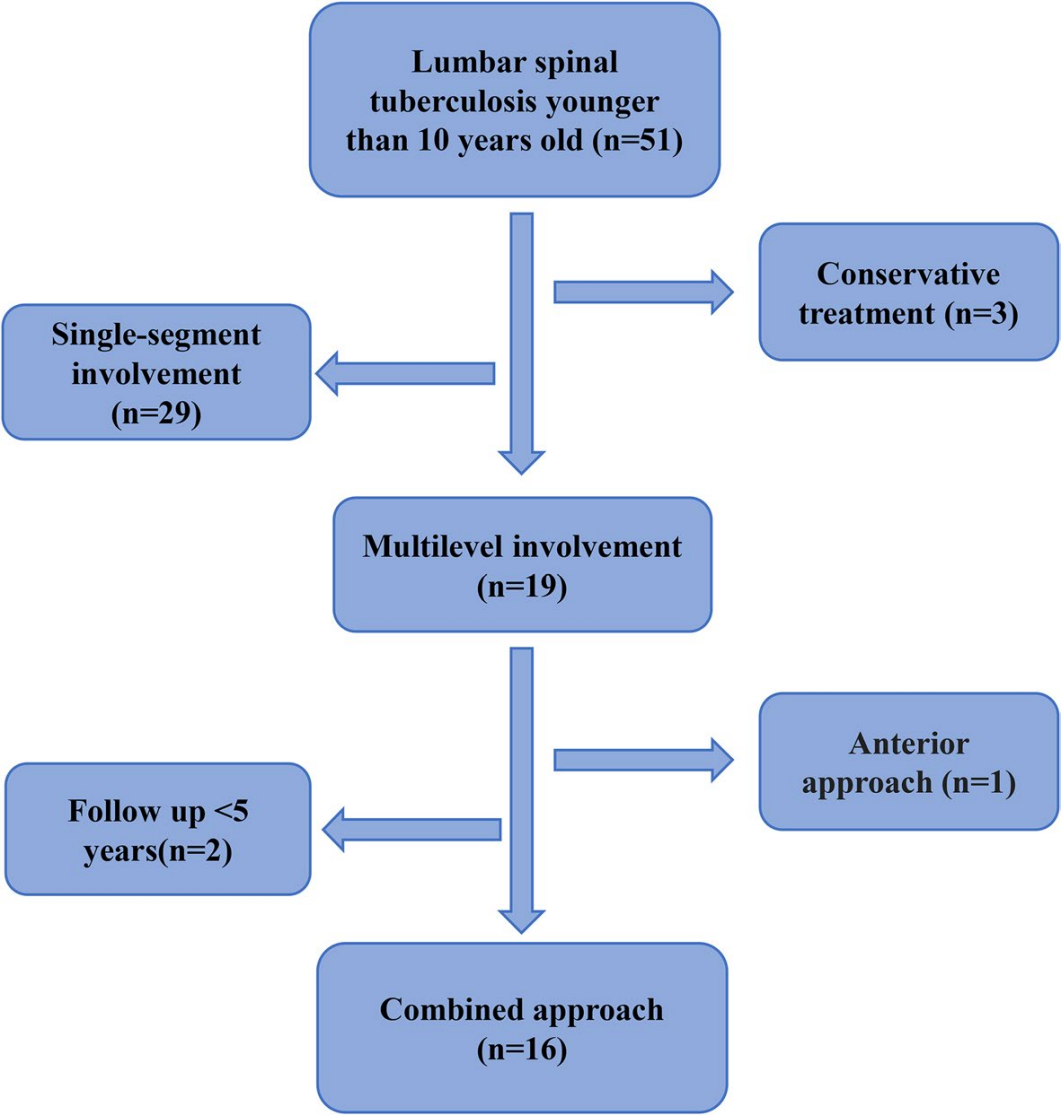
Flow chart of the study

Finally, 16 patients, consisting of 10 males and 6 females with an average age of 6.9 ± 2.2 years (3–10 years), were included in the analysis (Fig. 1). The involved vertebrae were observed at T12-L2 in three cases, L1-L3 in six cases, L2-L4 in five cases, and L3-L5 in two cases. Patients’ specific clinical characteristics are shown in Table 1.

**Table 1:** Patient basic demographic data

Variable	Results
Sex (M/F)	10/6
Age (years)	6.9 ± 2.2 (3–10)
≤ 5	5
>5	11
Body height (cm)	116.2 ± 13.6 (90.0–134.0)
Age ≤ 5 years	99.3 ± 6.5 (90.0–106.0)
Age>5 years	123.9 ± 7.3 (112.0–134.0)
Weight (kg)	21.7 ± 4.9 (13.5–28.2)
Age ≤ 5 years	15.7 ± 1.6 (13.5–17.5)
Age>5 years	24.4 ± 2.9 (19.0–28.2)
Affected vertebrae	
T12-L2	3
L1-L3	6
L2-L4	5
L3-L5	2
Abscess	
Left	5
Right	4
Both	7

*M* Male, *F* Female

This study was approved by the ethics committee of the Second Xiangya Hospital of Central South University, and signed informed consent was obtained from all guardians of patients.

### Preoperative treatment

Several clinical tests were completed, including chest X-ray, lumbar spine X-ray, computed tomography (CT), magnetic resonance imaging (MRI), sedimentation rate (ESR), C-reactive protein (CRP), purified protein derivative (PPD), TB antibody, and liver and kidney functions. All patients were administrated with oral anti-TB drugs (rifampicin 5–10 mg/kg/d, isoniazid 5–10 mg/kg/d, ethambutol 15 mg/kg/d, and pyrazinamide 20–30 mg/kg/d) for 2–4 weeks. Surgery was not performed until the ESR and CRP decreased significantly, the temperature returned to normal, and hypoalbuminemia and anemia were rectified. If the patient’s neurological deficits aggravated, emergency surgery was carried out.

### Surgical procedure

All procedures were performed by the same group of spinal surgeons in our department. The patient was positioned in the prone position following general anesthesia. The laminae and facet joints and the adjacent segments were exposed using subperiosteal dissection through a standard dorsal midline incision. Appropriate screw was selected based on patient’s age and transverse diameter of the pedicle on preoperative anteroposterior lumbar radiograph. Generally, 4.0-mm pedicle screw and 3.2/3.5-mm titanium rod was used for patients aged ≤5 years, and 5.0/5.5-mm pedicle screw and 4.75-mm CoCrMo/5.5-mm titanium rod was used for patients aged>5 years. Suitable pedicle screws were subsequently implanted into the two vertebrae above and below the affected segments, and short screws were inserted in the eroded vertebra if possible. Then, deformity correction was performed using a cantilever beam technique. Ponte osteotomies were done to increase flexibility when stiffness was found. After instrumentation and deformity correction, the laminae of the diseased segments were decorticated, and then an adequate amount of allogeneic cancellous bone strips were grafted.

The child was then placed in a lateral position, and a unilateral or bilateral abdominal incision was determined according to the site of the abscess and the destruction of the vertebrae. The lesion was exposed via the retroperitoneal space. The abscesses, necrotic bone, destroyed intervertebral discs, and granulation tissues were thoroughly debrided, and the epiphysis was preserved as much as possible. After adequate decompression of the spinal cord, a suitable freeze-dried allogenic iliac-derived strut bone (Allogenic Bone Grafts, OsteoRad, Taiyuan, Shanxi Province, China) was placed in the defect for reconstruction. Finally, a gelatin sponge containing 0.4–0.6 g amikacin and 0.2–0.3 g isoniazid was administered in the focus, and the incision was closed in layers after placing a drain.

### Postoperative management

The drainage tube was not removed until the volume was less than 30 ml/d. Patients underwent lumbar radiography 1 week postoperatively. Intravenous antibiotics were used for 2–3 days. Pyrazinamide was discontinued 2 months postoperatively, and administration of the remaining anti-TB drugs continued 12 months postoperatively. Hepatoprotective drugs were also administered. Patients could walk around under brace protection after 2 weeks till bony fusion was achieved.

### Follow-up and evaluation index

Regular follow-ups were carried out during which the lumbar spine X-ray and CT/MRI, if necessary, were performed. Blood routine, ESR, and CRP were used to detect the activity of TB at regular intervals. During chemotherapy, patients were also monitored for liver and renal functions. The patient’s neurological dysfunction was evaluated using the Frankel grade [8]. The grade of bony fusion was assessed according to the grading system [9]. Frankel grade, kyphosis Cobb angle, lumbar lordosis (LL), pelvic incidence (PI)-LL, ESR, and CRP were recorded during the follow-up. In addition, pain scales, including faces, legs, activity, cry and consolability (FLACC) when patients’ age ≤ 5 years, and visual analog scale (VAS) when patients’ age > 5 years, were also recorded.

### Statistical analysis

Statistical analysis was performed using SPSS 23.0 software (SPSS, Chicago, Illinois, USA). Continuous variables were expressed as mean ± standard deviation. Paired t-test was used to compare differences in ESR, CRP, and radiological parameters before and after surgery. Independent samples t-test was used for comparison of differences between groups. Wilcoxon signed-rank test was performed to compare changes in the Frankel grade during preoperative, postoperative, and final follow-up. *P* < 0.05 was considered statistically significant.

## Results

The average operation time was 275.3 ± 25.9 min, with 107.2 ± 19.1 and 168.1 ± 28.6 min for posterior and anterior surgery, respectively. The average blood loss was 334.1 ± 40.9 ml, with 180.9 ± 31.5 and 153.1 ± 31.2 ml for posterior and anterior surgery, respectively. No major vessel injury or dural tear occurred during the operation. A peritoneal tear occurred in one case and was repaired without any postoperative discomfort. Two patients had a superficial infection of the anterior wound, which healed well after antibiotic application and multiple dressing changes.

The average follow-up time was 7.8 ± 2.4 years. The mean pretreatment CRP and ESR were 47.7 ± 13.1 mg/L and 60.4 ± 10.3 mm/h, which decreased significantly to 7.7 ± 3.0 mg/L and 10.1 ± 3.3 mm/h 6 months postoperatively, respectively. CRP and ESR of all patients returned to the normal range within 1 year. All patients achieved significant improvement in pain symptoms after surgery compared to that of preoperative, with almost complete pain relief at the last follow-up. (Table 2).

**Table 2:** Perioperative and follow-up outcomes

Variable			Results
Operation time (min)			275.3 ± 25.9 (215–325)
Posterior surgery			107.2 ± 19.1 (80–150)
Anterior surgery			168.1 ± 28.6 (105–225)
Blood loss (ml)			334.1 ± 40.9 (260–405)
Posterior surgery			180.9 ± 31.5 (105–230)
Anterior surgery			153.1 ± 31.2 (90–210)
Follow-up time (years)			7.8 ± 2.4 (5–12)
Back pain score	Preoperative	Postoperative	Final follow-up
FLACC (age ≤ 5 years)	6.4 ± 1.1 (5–8)	2.8 ± 0.8 (2–4)^*^	
VAS (age>5 years)	5.8 ± 1.2 (4–8)	2.6 ± 1.3 (1–5)^*^	
VAS (all patients)			0.5 ± 0.6 (0–2)
Inflammatory indicator	Preoperative	Post-op 6 months	Post-op 1 year
ESR (mm/h)	60.4 ± 10.3 (42–76)	10.1 ± 3.3 (5–16)^*^	4.1 ± 1.9 (1–8)^**^
CRP (mg/L)	47.7 ± 13.1 (29–76)	7.7 ± 3.0 (3–13)^*^	3.5 ± 2.0 (0–7)^**^

*FLACC* Faces, legs, activity, cry and consolability, *VAS* Visual analog scale, *ESR* Erythrocyte sedimentation rate, *CRP* C-reactive protein

*: postoperative or post-op 6 months vs. preoperative *P*<0.01; **: post-op 1 year vs. preoperative *P*<0.01

No instrumentation failure or TB recurrence occurred among the patients. All patients achieved solid bone fusion at the last follow-up. No obvious bone graft displacement was found; however, one case showed slight subsidence of the graft during follow-up but the patient had no local pain or neurological dysfunction, and no apparent deformity was observed at the last follow-up.

The preoperative kyphosis angle was 29.9 ± 8.1°, which decreased significantly to 5.9 ± 2.6 postoperatively. There was a mild loss (2.5°) and the kyphosis angle was 8.4 ± 2.9° at final follow-up, with an overall correction rate of 71.3%. Postoperatively and at the last follow-up, all patients showed significant improvement in sagittal parameters (LL and PI-LL) compared to those of preoperative (Table 3). Furthermore, patients were divided into two groups (age ≤ 5 years and age>5 years). There were no significant differences between the two groups in terms of correction rate, correction loss rate and improvement in LL and PI-LL (Table 4).

**Table 3:** Preoperative and follow-up radiological parameters

Variable	Preoperative	Postoperative	Final follow-up
Kyphosis angle (°)	29.9 ± 8.1 (16.5–42.1)	5.9 ± 2.6 (1.0–11.0)^*^	8.4 ± 2.9 (2.0–13.2)^**^
Correction rate 1 (%)		80.3 ± 6.9 (71.0–96.3)	
Correction rate 2 (%)			71.3 ± 8.2 (55.1–94.7)
Correction loss rate (%)			9.0 ± 4.3 (1.6–19.4)
LL (°)	24.3 ± 4.9 (15.0–32.6)	38.4 ± 3.3 (32.0–43.0)^*^	42.2 ± 2.9 (35.9–45.7)^**^
PI-LL (°)	14.6 ± 3.8 (8.2–23.5)	0.5 ± 2.2 (−3.4–3.9)^*^	1.4 ± 2.1 (− 1.8–4.6)^**^

Correction rate 1 = %(preoperative-postoperative)/ preoperative; Correction rate 2 = %(preoperative-final follow-up)/preoperative; Correction loss rate = %(final follow-up- postoperative)/preoperative

*LL* Lordosis, *PI* Pelvic incidence

*: postoperative vs. preoperative *P*<0.001; **: final follow-up vs. preoperative *P*<0.001

**Table 4:** Radiological outcomes between patients with different ages

	Age ≤ 5 years (4.2 ± 0.8)	Age>5 years (8.2 ± 1.3)	*P* value
Follow-up time (years)	7.4 ± 2.3 (5–11)	8.0 ± 2.6 (5–12)	0.663
Kyphosis angle (°)			
Preoperative	30.1 ± 9.5 (17.6–41.3)	29.9 ± 7.8 (16.5–42.1)	0.966
Postoperative	5.5 ± 3.4 (1.4–11.0)	6.1 ± 2.3 (1.0–9.6)	0.711
Correction rate 1 (%)	80.5 ± 9.9 (71.0–96.3)	80.1 ± 5.7 (72.7–93.9)	0.920
Final follow-up	7.6 ± 4.0 (2.0–13.2)	8.7 ± 2.5 (4.2–12.1)	0.521
Correction rate 2 (%)	72.3 ± 14.3 (55.1–94.7)	70.8 ± 4.3 (63.6–76.2)	0.747
Correction loss rate (%)	8.2 ± 5.5 (1.6–15.9)	9.3 ± 4.0 (5.7–19.4)	0.610
LL (°)			
Preoperative	23.1 ± 4.8 (15.0–27.0)	24.9 ± 5.1 (17.0–32.6)	0.527
Postoperative	37.1 ± 3.6 (32.0–41.5)	39.0 ± 3.1 (32.5–43.0)	0.300
Final follow-up	41.0 ± 2.8 (38.5–44.5)	42.8 ± 2.9 (35.9–45.7)	0.264
PI-LL (°)			
Preoperative	14.8 ± 3.3 (11.8–20.0)	14.5 ± 4.2 (8.2–23.5)	0.895
Postoperative	0.7 ± 1.6 (−1.0–2.9)	0.3 ± 2.5 (−3.4–3.9)	0.739
Final follow-up	1.8 ± 1.1 (0.1–3.0)	1.2 ± 2.4 (−1.8–4.6)	0.482

Correction rate 1 = %(preoperative-postoperative)/preoperative; Correction rate 2 = %(preoperative-final follow-up)/preoperative; Correction loss rate = %(final follow-up- postoperative)/preoperative

*LL* Lordosis, *PI* Pelvic incidence

Exacerbation of neurological deficits was not observed in any patient. Compared with preoperative neurological deficits, postoperative and final follow-up neurological deficits improved significantly by grades 0.9 and 1.6, respectively. At the last follow-up, the neurological function of all patients recovered to grade E (Table 5).

**Table 5:** Preoperative and postoperative neurologic function

Grade	Preoperative	Postoperative	Final follow-up	*P*1	*P*2
A	0	0	0	0.001	0.001
B	3	1	0		
C	5	1	0		
D	7	7	0		
E	1	7	16		

*P*1, postoperative vs. preoperative, *P*2, final follow-up vs. preoperative

## Discussion

Pediatric spinal TB, characterized by fast bone destruction, is more likely to affect continuous multilevel vertebrae than adult spinal TB [10]. A previous study suggested that the average number of affected vertebrae and vertebral loss in patients younger than 10 years of age with lumbosacral TB was 1.6 and 2.5 times higher than in adults, respectively [4]. Besides, the pediatric spinal deformity may deteriorate with growth due to the imbalance in development between anterior and posterior parts of the spine even after the lesion is cured. Rajasekaran [11] found that age less than 10 years, vertebral body defect greater than 1–1.5, and pretreatment kyphosis angle greater than 30° were independent risk factors for severe progression. Furthermore, the spinal canal and nourishing vessels of the spinal cord are smaller in children than in adults, thus increasing the risk of neurological dysfunction in patients with spinal TB [12]. Severe kyphosis and neurologic deficits affect the quality of life of patients, which brings a heavy burden to their families and society. Thus, identifying an optimal procedure with the achievement of debridement, anterior column reconstruction, kyphosis correction, and deformity aggravation prevention for children with consecutive multilevel lumbar spinal TB is of great significance.

**Fig. 2:**
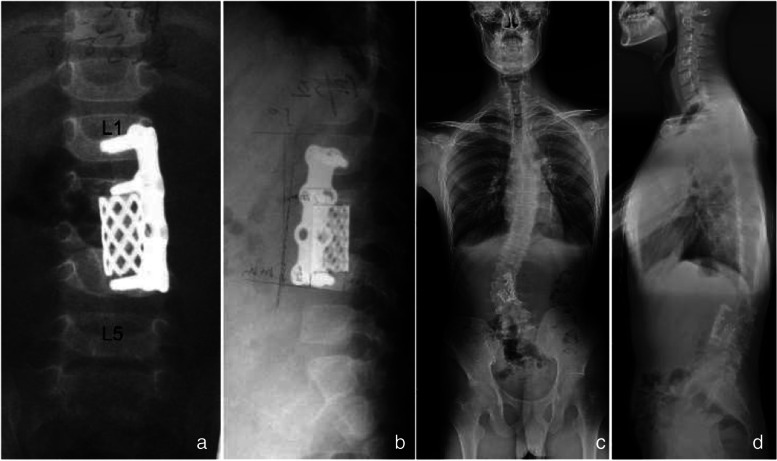
A 3-year-old boy with L2-L4 tuberculosis underwent anterior debridement and reconstruction using a titanium mesh and plate. Three-month postoperative X-ray showed good lumbar spine alignment and internal fixation position (**a**, **b**). Spinal deformity was observed at 15 years postoperatively (**c**, **d**)

Generally, tuberculous lesions are usually located in the anterior spinal column. The anterior approach facilitates lesion exposure and allows for debridement, decompression, and reconstruction under direct vision, which is a classic surgical procedure for the treatment of lumbar spinal TB [13]. However, this procedure has limited ability to correct the deformity [14]. Besides, anterior arthrodesis could decrease the ability for spinal self-shaping, thus failing to curb the progression of kyphosis, especially in cases of multi-segmental involvement [15]. Schulitz et al. [15] conducted a 5–10 years follow-up for 49 children with spinal TB who underwent anterior fusion and found that the kyphosis angle increased by an average of 12°. We performed a similar procedure on a child with multilevel lumbar TB and found that it could not effectively arrest the progression of the deformity due to the normal development of posterior structures (Fig. 2).

Anterior fusion with additional posterior instrumentation helps to solve the problem of correction loss by the equilibrating growth potential of the spine [7]. Moreover, robust internal fixation can provide sufficient stability, which is beneficial to healing spinal TB, obtaining bony fusion, and avoiding recurrence. Huang et al. [16] performed anterior fusion combined with posterior instrumentation in 15 patients aged 5–16 years with spinal TB and found that correction loss was only 4° at 30.3 months of follow-up. Notably, anterior debridement and reconstruction were implemented before posterior fixation in Huang et al.’s study. This procedure is not conducive to the correction of kyphosis and may increase the potential risk of graft displacement when the patient is transferred to the lateral position for posterior surgery [17]. Posterior correction followed by an anterior approach is preferred for pediatric multilevel lumbar TB with significant kyphosis and extensive abscesses. Elongation of a collapsed anterior column and optimal correction of kyphosis can be obtained by first performing posterior instrumentation, combined with Ponte osteotomies, if necessary. With the restoration of sagittal alignment, indirect decompression of the spinal cord can be achieved, followed by direct decompression through anterior debridement, which can further improve neurological function. Hu et al. [18] treated 20 cases of thoracolumbar TB in children using one-stage posterior instrumentation combined with anterior debridement and found that the average correction of kyphosis was 23.2° and significant improvement of neurological deficits was achieved 28.9 months postoperatively. This procedure was applied in the current study to treat 16 cases of pediatric multilevel lumbar spinal TB and found that the correction rate of kyphosis was 71.3% and neurological deficits of all patients returned to normal at the last follow-up.

The one-stage posterior approach is increasingly applied in the treatment of some selected spinal TB in adults and children. Zhang et al. [19] reported 22 children aged 4–16 years with monosegmental thoracolumbar TB treated using the posterior-only approach. At the final follow-up, the average correction of the kyphosis angle was 5.4° and neurological deficits achieved a significant improvement. However, the posterior-only approach may not be suitable for children with multilevel involvement and extensive abscesses since thorough debridement is critical for pediatric patients [3, 19–21]. Anterior column reconstruction via the posterior approach is more challenging and may carry a potential risk of nerve damage, especially in children with long-segment defects. Furthermore, when posterior normal structures are excised excessively without reliable reconstruction of the anterior column, the risk of postoperative pseudoarthrosis and instrumentation failure may increase. One could argue that the combined approach has the disadvantages of the long operation time, more blood loss, and extensive trauma. However, the integrity of posterior structures is largely preserved, and the lesion is exposed through the natural retroperitoneal space, thus not increasing the surgical trauma. In this study, the operation time and blood loss were 107.2 ± 19.1 min and 180.9 ± 31.5 ml and 168.1 ± 28.6 min and 153.1 ± 31.2 ml for posterior surgery and anterior surgery, respectively, showing satisfactory clinical results with no serious complications.

**Fig. 3:**
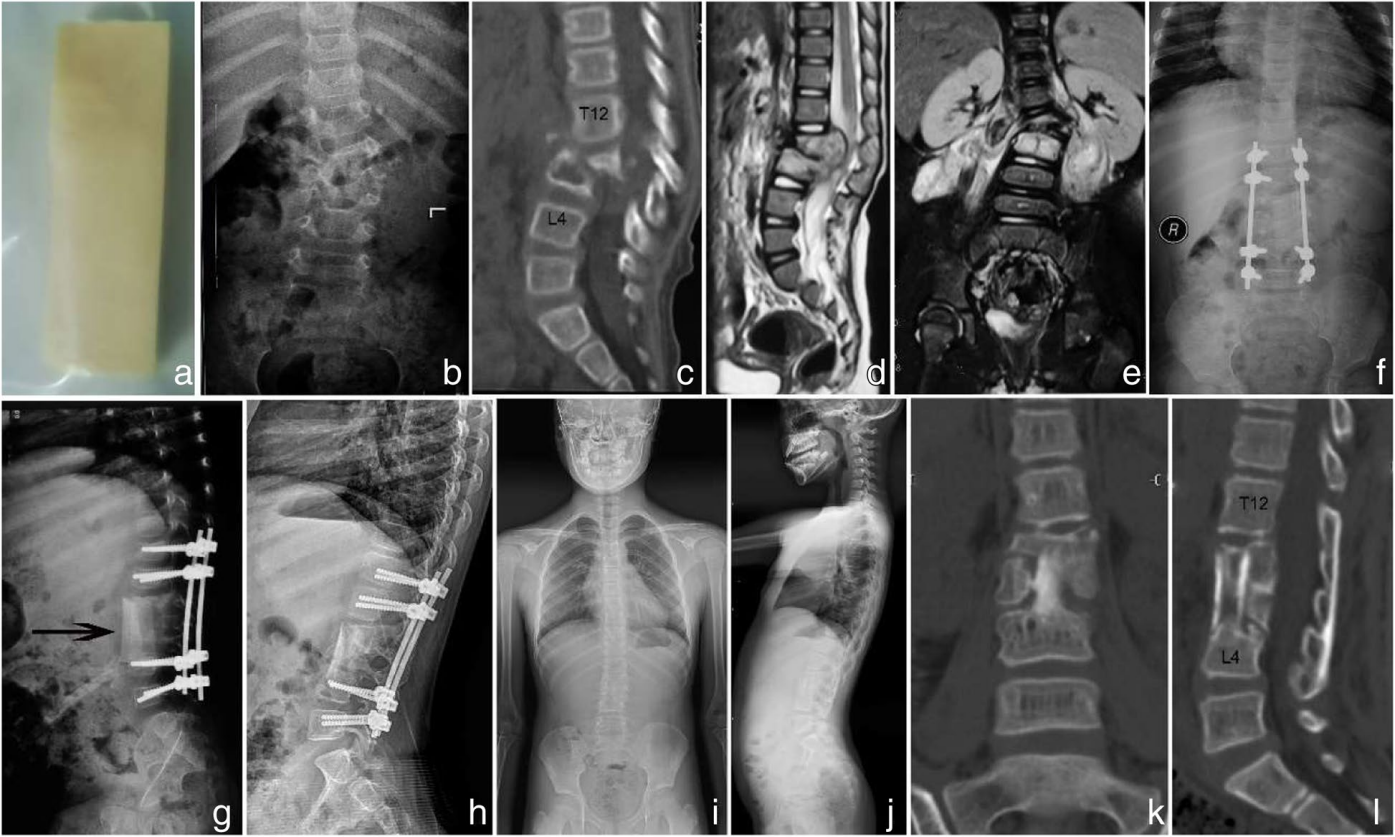
Allogenic strut bone (**a**). Preoperative imaging of a 3-year-old girl showing severe destruction of the vertebral body at L1-L3, with kyphosis, spinal cord compressed, and bilateral extensive abscesses (**b**-**e**). Posterior instrumentation combined with anterior debridement and reconstruction using allogenic strut bone was performed. Postoperative radiographs revealed perfect correction of kyphosis (**f**, **g**). Two-year postoperative X-ray showed a good position of the internal fixation, and the strut bone obtained reshape (**h**). Coronal and sagittal alignment of the patient were satisfactorily maintained 6 years postoperatively (**i**, **j**). Solid fusion was achieved (**k**, **l**)

Various structural graft materials such as autogenous iliac bone, rib, fibula, and titanium mesh have been used for anterior spinal column reconstruction. Autologous bone is considered the gold standard for the treatment of bone defects due to its high properties of osteogenesis, bone conductibility, and bone induction, as well as excellent biocompatibility [22]. However, the source and support strength of autologous strut bone for young children are limited [7]. Although titanium mesh cages filled with allogeneic and locally sourced autogenous bone are often used for anterior column reconstruction, they also have certain drawbacks. Because of its sharp edges, the titanium mesh cage may sink into the adjacent vertebral body, resulting in loss of kyphosis correction and reduction of intervertebral height [23]. Besides, titanium mesh can produce artifacts in MRI/CT due to its metallic properties, which is not conducive to the observation of fusion status and lesion activity during follow-up [24]. Zhang et al. [25] implemented anterior column reconstruction using fresh-frozen allograft in 14 children with single-segment lumbar spinal TB, and all patients achieved successful bone fusion. Allogenic strut bone was used in the current study to reconstruct multi-segmental lumbar spine defects and obtained satisfactory outcomes. The allograft is a commercialized bicortical bone (Fig. 3a), which is inexpensive, readily available, and easily stored. It is derived from human iliac and processed by sterilization, demineralization, lyophilization and irradiation, which greatly weakens the immunogenicity and reduces the risk of disease transmission while preserving the original mechanical properties of the bone. No negative effects on disease healing and loosening, dislocation, or fracture of the strut bone were observed, except for only one case with mild subsidence.

**Fig. 4:**
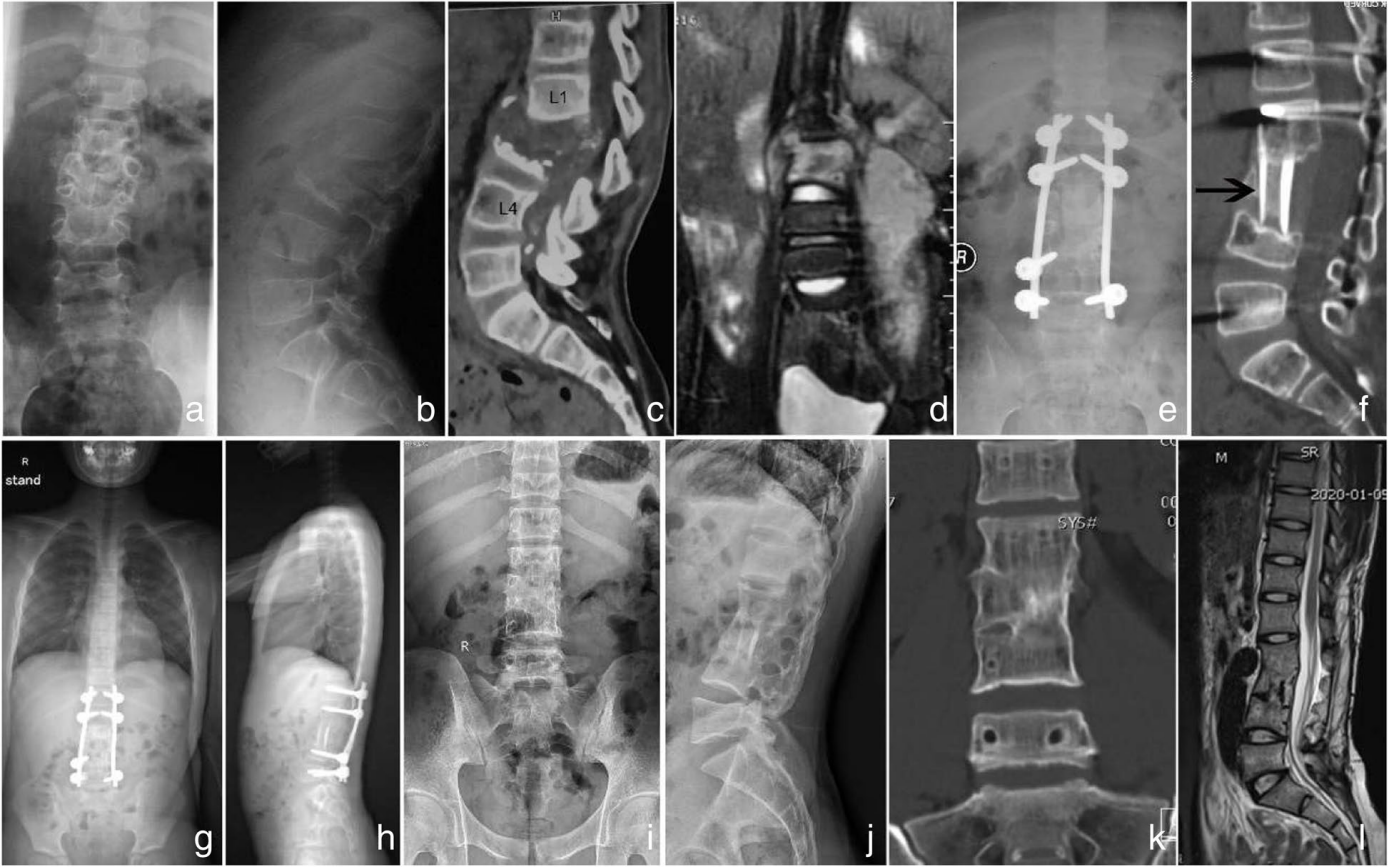
Preoperative imaging of a 7-year-old boy demonstrating L1-L3 involvement, with almost complete disappearance of L2 and L3 vertebral body resulting in spinal instability. In addition, dead bone fragments protruded into the spinal canal and there were extensive psoas abscesses (**a**-**d**). Three-month postoperative imaging showed satisfactory correction of kyphosis (**e**, **f**). Three-year postoperative X-ray showed that the position of the internal fixation and strut bone was appropriate, and the coronal and sagittal alignment was good (**g**, **h**). X-ray showed no obvious coronal or sagittal deformities 12 years postoperatively (**i**, **j**). Solid fusion was achieved (**k**). MRI revealed that the spinal canal was unobstructed and the spinal cord was not compressed (**l**)

The authors stated that the advantages of allogenic strut bone are multifold: avoiding trauma and donor-site complications associated with autologous bone harvest while reducing blood loss and saving operation time. The strut bone is available in different sizes: (10–80) mm*(5–40) mm *(5–30) mm, and can be trimmed to meet the needs of individualized treatment, depending on the intraoperative bone defect. It provides a sufficient contact area with endplates and has smooth edges and similar elasticity modulus to the vertebral body, which can effectively prevent postoperative subsidence. Moreover, the periphery of the allograft is rich in stiff cortical bone, thus providing enough bearing strength and creating stable biomechanics for the cure of lumbar spinal TB in children when combined with pedicle screws. Our follow-up results revealed that the strut bone has good biocompatibility and can be partially absorbed and well reshaped as the spine grows, eventually achieving a solid bone fusion (Figs. 3 and 4).

Although there is no definite evidence that screws negatively impact growth [26], we recommend removing posterior instrumentation after satisfactory fusion has been achieved, rather than until skeletal maturation, to avoid compromising the mobility of normal segments within the fixation zone and degeneration of adjacent segments.

This study has several limitations. This was a retrospective study with a limited sample size due to strict inclusion criteria. Besides, a case-control study with other procedures was not performed. Thus, a multicenter, large-sample randomized controlled trial should be carried out to further validate our results. Finally, spinopelvic parameters and their correlation with low back pain or health-related quality of life (HRQOL) outcomes have received considerable attention. In future relevant studies, whole spine X-rays should be performed and the association between sagittal parameters and HRQOL scores would be established.

## Conclusion

One-stage posterior instrumentation combined with anterior debridement and reconstruction using allogenic strut bone is a safe and effective procedure for the treatment of children with multilevel lumbar spinal TB. This approach facilitates removal of the lesion and decompression of the spinal cord and is effective in restoring spinal stability, correcting kyphosis, and preventing deterioration of the deformity.

## Data Availability

The datasets used and analysed in this study are available from the corresponding author on reasonable request.

## Abbreviations

TB: Tuberculosis
VAS: Visual analog scale
FLACC: Faces, legs, activity, cry and consolability
ESR: Erythrocyte sedimentation rate
CRP: C-reactive protein
PPD: Purified protein derivative
CT: Computed tomography
MRI: Magnetic resonance imaging
LL: Lumbar lordosis
PI: Pelvic incidence
F: Female
M: Male
HRQOL: Health-related quality of life
